# Draft Genome Sequence of Ureolytic Environmental Isolate *Bacillus galactosidilyticus* PL133

**DOI:** 10.1128/genomeA.01067-16

**Published:** 2016-10-06

**Authors:** Jonathan R. Gaiero, Philip A. Formusa, Tom Hsiang, Rob W. Nicol, Marc Habash

**Affiliations:** School of Environmental Sciences, University of Guelph, Guelph, Ontario, Canada

## Abstract

We report here the 5.19-Mb draft genome sequence of *Bacillus galactosidilyticus* PL133 isolated from poultry litter. The isolate was an important member of the cultivable aerobic bacteria identified to have ureolytic activity, which is responsible for ammonia generation in poultry litter residue.

## GENOME ANNOUNCEMENT

*Bacillus galactosidilyticus* PL133 was isolated in 2013 from a survey of cultivable aerobic bacteria present in poultry litter in southern Ontario, Canada (1). Isolate PL133 was identified as ureolytic based on a color change on Christensen’s urea agar indicator medium. PCR amplification and sequencing were performed on the alpha subunit (*ureC*) of the urease coding sequence (CDS), which was identified as closely matching the poultry litter urease producer (PLUP) group of urease sequences (2). This group, previously identified only by culture-independent sequencing, was found to be a dominant group in several poultry litters tested in the United States (2). Based on identification by 16S rRNA gene sequencing, it was determined to be an important member of the bacterial urease producers, comprising 14.3% of the community.

Genomic DNA was isolated from a pure culture grown in LB at 30°C. DNA was extracted using the DNeasy blood and tissue kit (Qiagen, Germantown, MD), according to the manufacturer’s recommended protocol for Gram-positive bacteria. Two micrograms of DNA was sent for sequencing using an Illumina HiSeq 2000 platform and 100-bp paired-end reads at the Génome Québec facility in Montreal, Quebec, Canada. A total of 23,912,403 reads were generated, with a genome sequence coverage of 902×. Genome assembly was performed using Velvet (version 1.2.10), generating 235 contigs. The draft genome comprised 144 contigs that were greater than 200 bp in length, with a total size 5,186,343 bp and an average G+C% content of 37.5%. The NCBI prokaryotic genome pipeline (GeneMarkS+ version 2.1) was used to predict a total of 2,458 protein-coding genes, nine rRNA genes, 62 tRNA genes, and 2,598 pseudogenes.

The scaffold assembly was also uploaded to the Rapid Annotations using Subsystems Technology (RAST) (3) website (http://rast.nmpdr.org/rast.cgi). As this is the first *Bacillus galactosidilyticus* strain to be sequenced, the closest neighbor was identified as *Bacillus coagulans* 36D1 (genome: 345219.8), with a score of 500. Identification of the urease (EC 3.5.1.5)-encoding operon (*ureABCDEFG*) supports the experimental evidence of ureolytic activity. The operon included genes encoding the alpha (*ureC*), beta (*ureB*), and gamma (*ureA*) subunits, as well as accessory proteins D, E, F, and G. Interestingly, there were two additional gene copies of *ureG* and one additional copy of *ureF*. Additionally, genes encoding an alternate pathway for urea degradation and ammonia generation, involving allophanate hydrolase (EC 3.5.1.54), were found. This included one gene copy of allophanate hydrolase subunit 1 and three copies of subunit 2. Other genes of interest include two copies of lanthionine biosynthesis (*lanL*), recombination repair (*ruvAB*), additional hypothetical (*yebC*), resistance to vancomycin (*vanW*), resistance to fosfomycin (*fosB*), resistance to beta-lactam (class A, *BLA*; class C, *BLc*), resistance mutations to fluoroquinolones (*parC*, *parE*, *gyrA*, and *gyrB*), and multidrug resistance efflux pumps (MATE family). Many resistance genes were unique to this isolate compared to other *Bacillus* spp., such as *B. cereus* and *B. amyloliquefaciens* via RAST. The presence of these resistance genes and genes relating to nutrient acquisition and stress response are likely resulting from the particular poultry litter environment in which they reside.

### Accession number(s).

This whole-genome shotgun project has been deposited at DDBJ/EMBL/GenBank under the accession no. LGPB00000000. The version described in this paper is the first version, LGPB01000000.
